# Targeting the indoleamine 2,3-dioxygenase pathway in cancer

**DOI:** 10.1186/s40425-015-0094-9

**Published:** 2015-12-15

**Authors:** Yong Wha Moon, Joud Hajjar, Patrick Hwu, Aung Naing

**Affiliations:** Medical Oncology, Department of Internal Medicine, CHA Bundang Medical Center, CHA University, Seongnam-si, Gyeonggi-do 463-712 South Korea; Department of Investigational Cancer Therapeutics, The University of Texas MD Anderson Cancer Center, 1515 Holcombe Blvd, Box 455, Houston, TX 77030 USA; Section of Immunology, Allergy & Rheumatology, Baylor College of Medicine, Texas Children’s Hospital, Houston, TX 77030 USA; Department of Melanoma Medical Oncology, The University of Texas MD Anderson Cancer Center, Houston, TX 77030 USA

**Keywords:** Indoleamine 2,3-dioxygenase, IDO inhibitors, Immune surveillance, Immunomodulatory, Malignancy

## Abstract

Tumor cells escape the immune surveillance system of the host through a process called immune tolerance. Immunotherapy targets molecules that serve as checks and balances in the regulation of immune response. Indoleamine-2,3-dioxygenase (IDO) is an intracellular enzyme, which through the process of tryptophan depletion exerts an immunosuppressive effect, facilitating immune escape of tumors. This review summarizes our current knowledge on IDO expression in malignancies, the IDO inhibitors that are currently available and those under clinical development.

## Introduction

Most tumors express potentially immunogenic antigens to which the immune system can respond [1]. In turn, the tumor-bearing host possesses high-avidity T cells that are specific to these antigens [2]. And yet, in a phenomenon called immune tolerance, tumor cells evolve to escape immune surveillance [3], and the host fails to reject the tumor. A number of molecular mechanisms underlying this induced tolerance have been identified [4]. Immunotherapies targeting such mechanisms have become an important therapeutic strategy for harnessing the immune system’s ability to control malignancy, overcoming immune tolerance.

Indoleamine-2,3-dioxygenase (IDO) is an intracellular heme-containing enzyme that initiates the first and rate-limiting step of tryptophan degradation along the kynurenine pathway [5]. In mammalian organisms, tryptophan is an essential amino acid for cell survival; it cannot be synthesized de novo. Initially, the role of IDO was thought to be mainly antimicrobial by reducing the availability of tryptophan in the inflammatory environment [6–8]. IDO was shown to be expressed in normal tissues such as the endothelial cells in the placenta and lung, the epithelial cells in the female genital tract, and the lymphoid tissues in mature dendritic cells (DCs) [9]. An innovative discovery by Munn and colleagues showed that IDO has a central role in preventing T cell-driven rejection of allogeneic fetuses during pregnancy as trophoblast expressing IDO was found to induce maternal tolerance to fetal allograft [10]. This discovery broke ground for further research addressing the immunomodulatory potential of IDO, including a series of studies focused on the role of IDO in the immune escape of tumors, reviewed earlier by Zou [4]. The immunosuppressive roles of IDO have also been investigated for elucidation of therapeutic targets in the management of autoimmune diseases [11] and for induction of graft tolerance after transplantation [12–15].

Several reviews discussing the enzymatic activity of IDO have been published [16–22]. The aims of this article are however, to summarize the most recent data on IDO expression in malignancies and to discuss the current status of clinical testing of IDO inhibitors.

## Review

### Mechanisms of IDO pathway involvement in immune tolerance

IDO is an important enzyme in both cancer development and cancer progression. IDO supports inflammation in the tumor microenvironment, development of immune tolerance to tumor antigens in stromal and immune cells, suppression of T and natural killer cells, generation and activation of T regulatory cells (Tregs) and myeloid-derived suppressor cells, and promotion of tumor angiogenesis [23].

Growing tumors evade the immune system of the host through a complex process of immunoediting in the tumor, where selective pressures result in the outgrowth of tumor clones capable of evading and suppressing active immunity, which often includes expression of tolerance mechanisms. The IDO pathway is one of the endogenous pathways used often by tumor cells to induce tolerance to tumor antigens [21].

**Fig. 1:**
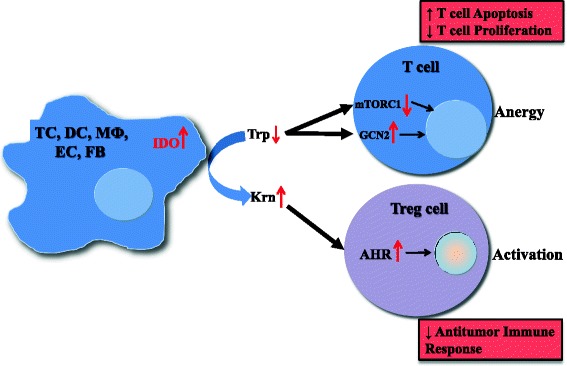
Mechanisms of IDO pathway activity in immune tolerance. The enzyme IDO catalyzes the initial and rate-limiting step in the catabolism of tryptophan along the kynurenine pathway. Accordingly, IDO can provoke tryptophan shortage, which results in mTORC1 inhibition and GCN2 activation, in turn leading to anergy of effector T cells. Tryptophan’s degradation leads to production of bioactive kynurenine pathway compounds, which activate the AHR, resulting in promotion of Treg differentiation. TC, tumor cell; DC, dendritic cell; MФ, macrophage; EC, endothelial cell; FB, fibroblast; IDO, indoleamine-2,3-dioxygenase 1; Trp, tryptophan; Krn, kynurenine; mTORC1, mechanistic target of rapamycin (serine/threonine kinase) complex 1; GCN2, general control nondepressible-2; AHR, aryl hydrocarbon receptor; Treg, regulatory T cell

As noted above, IDO is expressed by endothelial cells [24], mesenchymal stromal cells, fibroblasts [25], and various myeloid-derived antigen-presenting cells such as DCs and macrophages [26], as well as by tumor cells [23, 27]. However, some discrepancies in IDO expression profiles in normal and tumor tissues as well as cell types expressing IDO exist. For example, high expression of IDO in tumor draining lymph nodes (TDLN) was associated with poor clinical outcomes [28, 29]. However, recently it has been reported that TDLNs do not contain more IDO+ cells than normal lymph nodes in a study conducted in 15 patients with breast cancer and 15 patients with melanoma [9]. Similarly, multi-fold increase in expression of IDO was reported with mature DCs but not in immature DCs [9, 30]. On the contrary, some studies have reported expression of IDO1 by immature DCs or plasmacytoid DCs [28, 29]. Despite these discrepancies, the expression of IDO by antigen-presenting cells can lead directly to suppression of tumor-specific T-cell responses [27] and to activation of Tregs [31]. These effects can be achieved via multiple mechanisms, as demonstrated in Fig. 1.

An important mechanism by which IDO affects T-cell activity is that the consumption of local tryptophan inhibits mechanistic target of rapamycin (serine/threonine kinase) complex 1 (mTORC1) [32] as well as the T-cell receptor regulatory kinase, protein kinase C theta (PKC-θ) [32], both of which are regulatory targets of the master amino acid-sensing kinase, glucokinase (GLK1) [33]. mTORC1 inhibition can trigger a response that includes activating autophagy, leading to anergy in T cells in the tumor microenvironment [21].

IDO expression can further induce a stress response in cells via activation of general control nondepressible-2 (GCN2). Tryptophan degradation by IDO causes local tryptophan deficiency, leading to the accumulation of uncharged tryptophan transfer ribonucleic acid (tRNA) in cells. GCN2, a stress-response kinase, is stimulated by elevations in uncharged tRNA, and its activation can limit or alter protein translation [34] and prevent T-cell activation [35, 36]. Furthermore, activation of GCN2 promotes de novo Treg differentiation and enhances Treg activity, resulting in profound immunosuppressive microenvironment [36, 37].

An additional mechanism by which IDO expression alters the tumor microenvironment and favors immune tolerance is through the production of bioactive compounds. One such compound, kynurenine, which is produced by enzymatic activity of IDO on tryptophan, activates transcription of the aryl hydrocarbon receptor (AHR). Through the AHR, kynurenine metabolites promote differentiation of forkhead box P3-positive (FOXP3^+^) Tregs [38] and suppression of antitumor immune responses [39]. All three effects of AHR pathway signaling lead to anergy of effector T cells and activation of Tregs.

Sharma, Munn and colleagues showed that in melanoma mouse models, IDO is expressed by DCs in the tumor-draining lymph nodes (where tumors are exposed to the antigen-presenting cells for the first time), leading to potent suppression of cluster of differentiation (CD)8^+^ T-cell responses and to systemic tumor tolerance [40–42]. Those DCs, when harvested from the tumor-draining nodes and adoptively transferred into new hosts, led to suppression of T-cell responses *in vitro*, as well as antigen-specific T-cell anergy *in vivo* [29, 43]. In mouse models, IDO-expressing DCs activated Tregs, which then systemically suppressed antitumor T-cell responses [37]. The increases in Treg activity are achieved by activation of existing natural Tregs and by upregulation of FOXP3 expression in CD4b^+^ T cells so that they differentiate into new inducible Tregs [37, 42, 44]. Similar studies showed that IDO-expressing plasmacytoid DCs [45, 46] and IDO-expressing monocyte-derived (myeloid) DCs [22] induced differentiation of CD4^+^ cells into FOXP3^+^ Treg-like cells.

### Role of the IDO pathway in cancers

Aberrant IDO activity has been associated with a wide variety of non-oncologic human pathological conditions, including autoimmune diseases, infectious diseases, depression, obesity, organ and bone marrow transplantation and atherosclerosis. Direct evidence of IDO’s role has been obtained by studying patients, as well as relevant animal models [47–52].

Several lines of *in vitro* [53, 54] and *in vivo* [27, 29] evidence suggest that the IDO pathway plays a key role in regulating immune evasion by tumors. Recent evidence has demonstrated that the functionally active IDO protein is expressed in a wide variety of human hematologic malignancies, such as acute monocytic leukemia, [55] acute lymphocytic leukemia, [55] acute myeloid leukemia [56] and T-cell leukemia/lymphoma [27], and solid tumors, such as breast cancer [27, 57], colorectal cancer [27], endometrial cancer [27], gastric cancer [27], glioblastoma [27], gynecological cancers [58], head and neck cancers [27], non-small cell lung cancer (NSCLC) [27], small cell lung cancer [27], melanoma [27], mesothelioma [27], and pancreatic cancer [27]. In contrast, most normal cells of the stroma were found to be negative for IDO expression [27]. Several studies have attempted to link IDO activity with its proposed mechanism of action, demonstrating that IDO expression/activity is associated with reduced intratumoral T-cell infiltration, disease progression, and decreased shorter overall survival. For example, an increased kynurenine/tryptophan ratio in the blood was associated with a shorter survival time in patients with acute myeloid leukemia [59]. In patients with solid tumors, such as colorectal cancer [60, 61], endometrial cancer [62, 63], small cell lung cancer [64], melanoma [65], and ovarian cancer [66, 67], high IDO expression is correlated with a poor prognosis and shorter overall survival. The shorter survival of patients whose tumors overexpress IDO supports the concept that a treatment strategy of IDO blockade will have antitumor effects. IDO is thus an attractive target for therapeutic intervention.

IDO may also be involved in mechanisms leading to chemoresistance in cancer patients. In a study of gene expression profiling associated with paclitaxel resistance in patients with serous ovarian cancer, *IDO* was the most prominently expressed gene. This finding was confirmed with real-time reverse transcription-polymerase chain reaction and immunohistochemistry [67].

Furthermore, the regulation of IDO was demonstrated to contribute substantially to the antitumor effects of imatinib in a mouse model of spontaneous gastrointestinal stromal tumor [68]. Imatinib activated CD8^+^ T cells and induced Treg apoptosis within the tumor by reducing tumor cell expression of IDO [68]. It is speculated that concomitant immunotherapy with an IDO inhibitor may further improve outcomes in gastrointestinal stromal tumor treated with imatinib.

### IDO inhibition as a therapeutic strategy

There has been increasing scientific interest in IDO as a novel therapeutic target for the development of new cancer drugs, based on the *in vitro* and *in vivo* evidence for immune tolerance in the setting of IDO expression in tumor cells and the clinical evidence for poor prognosis and chemoresistance in tumors with high IDO expression. Indeed, potential IDO-inhibiting drugs for use in human cancers are now the focus of research and development efforts. Current inhibitors of IDO are listed in Table 1.

**Table 1:** Reported IDO inhibitors

Agent	Mechanism	First author, year
D-1MT	Tryptophan mimetic; D isoform of MT; Transcriptional suppressor of IDO	Metz 2012 [32]
L-1MT	Tryptophan mimetic; L isoform of MT; selective IDO1 inhibitor	Opitz 201 [39]
MTH-Trp	Tryptophan mimetic; transcriptional suppressor of IDO	Okamoto 2007 [92]
β-carbolines	Tryptophan mimetic; IDO and TDO inhibitor	Eguchi 1984 [93]
Naphthoquinone-based	Pharmacophore of natural product annulin B; indole mimetic; IDO inhibitor	Kumar 2008 [94]
S-allyl-brassinin	Phytoalexin; indole mimetic	Gaspari 2006 [95]
S-benzyl-brassinin	Phytoalexin; indole mimetic	Gaspari 2006 [95]
5-Bromo-brassinin	Phytoalexin; indole mimetic	Banerjee 2008 [96]
Phenylimidazole-based	Computationally designed synthetic IDO inhibitor	Kumar 2008 [94]
4-phenylimidazole	Heme ligand in IDO enzyme	Sono 1989 [97]
Exiguamine A	Non-tryptophan analogue	Harry 2006 [98]
NSC401366	Non-indolic IDO inhibitor	Vottero 2006 [99]

*IDO* Indoleamine-2,3-dioxygenase 1, *1MT* 1-methyl-DL-tryptophan, *MTH-Trp* methylthiohydantoin-dl-tryptophan, *TDO* tryptophan-2,3-dioxygenase

Among the IDO inhibitors, 1-methyl-DL-tryptophan (1-MT) has been the most widely studied. There are 2 available stereoisomers of 1-MT, D and L isomers, with potentially different biochemical and antitumor activity [69]. Pioneering work performed by Hou et al revealed cell-type specific variations in the activity of the 1-MT isomers. In *in-vitro* studies, the L isomer (L-1MT) is superior in inhibiting the enzymatic activity of IDO (kynurenine production from tryptophan) in cell-free assays, and several cell lines [69]. Nevertheless, D isomer (D-1MT) is equally effective in inhibiting the enzymatic activity of IDO in human monocyte-derived DCs in allogeneic mixed lymphocyte reactions (MLRs) and is significantly superior to L-1MT or DL mixture in inducing T-cell proliferation in allo-MLRs using either human T cells stimulated by IDO-expressing monocyte-derived DCs or murine T cells stimulated by IDO-expressing plasmacytoid DCs from tumor-draining lymph nodes [69]. However, this effect is not observed in MLRs using IDO-KO DCs, indicating that the D-1MT exerted its effect in allo-MLRs directly by its action on the *IDO* gene [69]. Hou et al attributed the cell-type specific variations in the activity of the 1-MT isomers to the possible existence of IDO isoforms. In addition, a statistically significant prolongation of survival is observed with D-1MT in combination with cyclophosphamide in orthotopic 4 T1-luc tumors and in combination with paclitaxel in the autochthonous MMTV-Neu breast tumor model, compared to L-1MT [69]. The superiority of one isomer over the other is therefore dependent on the biological context of IDO expression, tumor cells versus host dendritic cells. Based on the above findings, D-1MT has been considered more suitable for further clinical development. Soon after, Metz et al reported the discovery of a novel IDO-related tryptophan-catabolizing enzyme, IDO2 that is coded by a gene, *IDO2*, upstream of the *INDO* gene that codes for IDO (dubbed IDO1). Though IDO2 shares structural similarity to IDO1 [70], IDO2 is expressed only in a subset of tissues that express IDO1 but includes DCs [71]. Consistent with the observations made by Hou et al, Metz et al demonstrated that L-1MT modestly inhibits IDO1 activity, whereas D-1MT inhibits IDO2. This possibly explains the greater antitumor activity of D-1MT in preclinical models. Despite the superior antitumor activity of D-1MT, the response to treatment may be negated by the existence of functionally inactive polymorphisms in the *IDO2* gene that ablate the enzymatic activity in approximately 50 % of white populations and 25 % of individuals of African descent [71]. The genetic status of the patient regarding *IDO2* gene may serve as a predictive marker of response.

Contrary to these findings, Lob et al reported that D-1MT may not be an effective therapeutic option based on a study in a subset of human DCs [72]. Following treatment of interferon-γ-treated mature DCs with *IDO1*-specific siRNA, IDO1 transcription was silenced; while expression of IDO2 was unaffected. In addition, tryptophan and kynurenine concentrations were restored following treatment with *IDO1*-specific siRNA. Similar results were observed in tumor samples that were known to constitutively express IDO [73]. Based on these findings, Lob et al concluded that the IDO activity is only due to IDO1; and IDO2 though expressed by human DCs, was functionally inactive. Therefore inhibition of IDO activity in human DCs by D-1MT was considered unlikely, despite its activity in mice [73, 74]. More recently, genetic ablation of IDO1 alone was sufficient to phenocopy IDO‐inhibition with either D‐1MT or combined D- and L-1MT regarding important biomarkers of immune activation after chemo‐radiation therapy in a mouse brain tumor model [75]. Based on this finding therapeutic inhibition of IDO pathway largely directed against IDO1 has been explored [76]. Despite the inability or poor ability to inhibit the enzymatic activity of IDO, D-1MT exhibits superior antitumor activity. It is speculated that it interferes with IDO function at other levels besides its inhibitory action on IDO2 [32]. An incidental finding that D-1MT acts as a potent tryptophan mimetic in mTOR regulation by restoration of mTOR pathway is one such possible mechanism of action [32]. Further, IDO-mediated catabolism of tryptophan also inhibits the immunoregulatory kinases mTOR and PKC-θ, in addition to inducing autophagy. These effects were also relieved specifically by tryptophan and D-1MT [32]. On the contrary, the immunostimulatory effect of L-1MT is limited by activation of the AHR pathway in response to production of N-methyl-kynurenine, a metabolite of L-1MT. These factors help to explain why L-1MT is considered a poor physiological inhibitor of IDO in comparison to D-1MT and why D-1MT has broader clinical uses against cancers that overexpress any tryptophan catabolic enzyme, such as IDO1, IDO2 and tryptophan-2,3-dioxygenase (TDO) [32].

### IDO inhibitors under clinical development

Currently, four IDO inhibitors are under clinical development (Table 2): INCB024360 (developer: Incyte) [76, 77], indoximod (D-1MT; developer: NewLink Genetics) [32], an IDO peptide vaccine (developer: Copenhagen University) [78], and NLG919 (developer: NewLink Genetics, recently licensed to Genentech) [79].

**Table 2:** Clinical trials using IDO inhibitors

Drug	Phase	Mono or combo	Cancer	Clinical trial ID	Comments
INCB 024360	I	Mono	All	NCT01195311	ASCO 2013
	Ib/II	With ipilimumab	Melanoma	NCT01604889	ASCO 2014/Recruiting
	II	With MELITAC 12.1	Melanoma	NCT01961115	Recruiting
	II	Mono	Ovary	NCT01685255	Recruiting
	II	Mono	MDS	NCT01822691	Recruiting
	Ib/II	With MK3475	All/NSCLC	NCT02178722	Recruiting
	Ib/II	With CDX-1401, poly ICLC	Ovary	NCT02166905	Recruiting
	Pilot	Mono	Ovary (neoaduvant)	NCT02042430	Recruiting
Indoximod (NLG2101)	I	Mono	All	NCT00739609	ASCO 2012
	Ib	With docetaxel	All	NCT01191216	ASCO 2013
	Ib/II	With AD.p53 DC vaccine	All/breast	NCT01042535	ASCO 2013/Recruiting
	II	With docetaxel	Breast (HER2-)	NCT01792050	Recruiting
	II	Mono	Prostate	NCT01560923	Recruiting
	II	With sipuleucel-T	Prostate	NCT01560923	Recruiting
	Ib/II	With nab-paclitaxel, gemcitabine	Pancreas	NCT02077881	Recruiting
	Ib/II	With ipilimumab	Melanoma	NCT02073123	Recruiting
	Ib/II	With temozolomide	Brain	NCT02052648	Recruiting
IDO peptide vaccine	I	With imiquimod, montanide	NSCLC	NCT01219348	Iversen 2013 [78]
	Ib	With temozolomide	Melanoma	NCT01543464	Recruiting
NLG919	I	Mono	All	NCT02048709	Recruiting

*DC* dendritic cell, *MDS* myelodysplastic syndrome, *NSCLC* non-small cell lung cancer

INCB024360 is an orally administered hydroxyamidine small molecule inhibitor of IDO1. The findings of a phase I study of this IDO inhibitor were reported during a poster discussion session at the 2013 American Society of Clinical Oncology (ASCO) annual meeting [80]. This study was an open-label, single-agent dose-escalation trial in 52 patients with advanced malignancies (NCT01195311). Using two independent pharmacodynamic assays, IDO1 inhibition was observed in all patients receiving the compound. Treatment with ≥ 300 mg INCB024360 twice a day resulted in greater than 90 % inhibition of IDO1 activity, and the drug was well tolerated. The most common grade 1 or 2 adverse events were fatigue and gastrointestinal disturbances, and the most common grade 3 or 4 adverse events were abdominal pain, hypokalemia and fatigue. The maximum tolerated dose was not established, but based on the pharmacokinetic and pharmacodynamic data indicating that prespecified target inhibition was achieved and on the available tablet strengths, the recommended dose for a phase II study was 600 mg twice daily. The compound led to stable disease for greater than 8 weeks in approximately 30 % of these patients with highly refractory disease; half of the patients had colorectal cancer, which has been refractory to most immunotherapy approaches. Together, these results suggest that INCB024360 has the potential to be effective as monotherapy, but, since it was so well tolerated, it may be particularly useful in combination with other cancer agents. Currently, two phase II trials of INCB024360 monotherapy are recruiting patients with ovarian cancer (NCT01685255) or myelodysplastic syndrome (NCT01822691). Furthermore, INCB024360 monotherapy is being tested in a neoadjuvant pilot study in ovarian cancer (NCT02042430).

Combining INCB024360 and other immunotherapy modalities such as a cancer vaccine or immune checkpoint inhibitor is being actively investigated based on preclinical data showing synergism of IDO inhibitors and other immune checkpoint blockade strategies [81, 82]. In a phase Ib study of INCB024360 in combination with ipilimumab in patients with advanced melanoma (NCT01604889), which was presented at the 2014 ASCO annual meeting [83], tumor response and duration data suggested the potential for enhanced outcomes with combination treatment compared to ipilimumab monotherapy in melanoma patients. Furthermore, a phase Ib/II study of INCB024360 in combination with another immune checkpoint inhibitor, MK3475, which is a programmed cell death-1 (PD-1) inhibitor, has just started (NCT02178722). Two clinical trials of INCB024360 combined with cancer vaccines are recruiting patients: a phase II pilot study in combination with MELITAC 12.1, which is a multipeptide melanoma vaccine (NCT01961115); and a phase Ib/II study in combination with both CDX-140 [84], which is a vaccine for tumor antigen NY-ESO-1, and poly ICLC [84], which is a viral mimic composed of stabilized double-stranded RNA that acts as a ligand to Toll-like receptor 3 (NCT02166905).

Indoximod (NLG2101), which is D-1MT, is an orally administered IDO pathway inhibitor. An initial phase I study of indoximod as a single agent demonstrated good oral bioavailability and a favorable safety profile [85]. Based on these findings, a phase Ib trial of indoximod in combination with docetaxel (NCT01191216) was conducted in 27 patients; the results were reported at the ASCO annual meeting in 2013 [86]. The data showed that among 22 evaluable patients, 18 % (4/22; 2 with breast cancer; 1, NSCLC; 1, thymus cancer) exhibited a partial response, and 41 % (9/22; 4 with breast cancer; 2, NSCLC; 1, laryngeal cancer; 1, esophageal cancer; 1, ovarian cancer) had stable disease. Data indicated that the combination therapy was well tolerated with no increase in toxicities over those expected for the individual agents and no unexpected drug-drug interactions. Further, the pharmacokinetic profile of the combination therapy was similar to the profile of each drug as a single agent. Based on these results, a randomized phase II trial was initiated to evaluate the potential of indoximod in combination with docetaxel in patients with metastatic breast cancer (NCT01792050). Two more phase Ib/II trials of combined indoximod and chemotherapy are being conducted; indoximod plus albumin-bound paclitaxel (nab-paclitaxel) and gemcitabine in pancreatic cancer (NCT0277881) and indoximod plus temozolomide in brain tumors (NCT02052648).

Indoximod is also being tested in combination with other immunotherapy, such as a cancer vaccine or immune checkpoint inhibitor. First, a phase Ib study of combined indoximod and DC cancer vaccine (AD.p53DC) was presented at the ASCO annual meeting in 2013 [87]. The combination was well tolerated with no dose-limiting toxicities. The safety profile was consistent with the known monotherapy safety profiles for each agent. Among 32 patients enrolled in the phase I study, treatment discontinuations were all due to disease progression, not due to toxicities. No objective responses to the study therapy were noted, but after subsequent post-vaccine chemotherapy, 6 of 10 breast cancer patients treated with carboplatin/gemcitabine or gemcitabine alone showed response. This finding supports the hypothesis that immunotherapy treatments may sensitize patients to subsequent lines of therapy. Currently, a phase II trial is looking at the response to indoximod plus AD.p53DC vaccine followed by salvage carboplatin/gemcitabine in previously treated breast cancer patients (NCT01042535).

A second combination of indoximod with immunotherapy--a randomized, double-blind, phase II study of indoximod or placebo following infusion of sipuleucel-T--is being conducted in men with asymptomatic or minimally symptomatic metastatic castration-resistant prostate cancer (NCT01560923). Finally, another phase Ib/II study of combined indoximod and ipilimumab is recruiting patients with melanoma (NCT02073123).

Data from a phase I trial of an IDO peptide vaccine in 15 HLA-A2-positive patients with stage III-IV NSCLC were recently published [78]. The vaccine was well tolerated, with no severe toxicity. One patient developed a partial response, and 6 patients (40 %) demonstrated stable disease for ≥ 8.5 months [78]. Meanwhile, a phase Ib study of combined IDO peptide vaccine and temozolomide is under way (NCT01543464).

NLG919 is the most recently developed orally bioavailable IDO1 inhibitor [79]. It was developed by the same company that developed the IDO pathway inhibitor indoximod. In preclinical setting, NLG919 was found to be as effective as indoximod in enhancing the survival in mice with glioblastoma and was synergistic when combined with standard chemo-radiation therapy [75]. Based on this preclinical finding, a phase I dose-escalating study is recruiting patients with advanced solid tumors (NCT02048709).

### Future directions

**Fig. 2:**
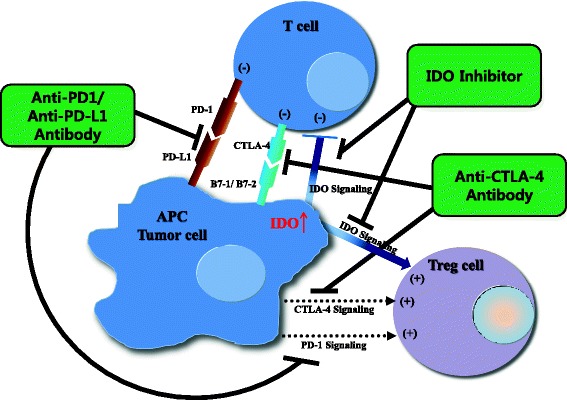
Potential mechanism of combinatory synergism between three methods of immune checkpoint blockade. PD-1 or CTLA4 signaling inhibits proliferation of effector T cells. In addition, some studies have suggested that Tregs are also partly activated by PD-1 or CTLA4 signaling [100]. IDO signaling induces tumor immune tolerance by both suppressing effector T cells and activating Tregs. Therefore, if PD-1 or CTLA-4 signaling blockade is combined with an IDO inhibitor, effector T cells may be further activated and Tregs may be further suppressed. The result could be more effective reversal of immune tolerance and enhanced anti-tumor immune response. APC, antigen-presenting cell; PD-1, programmed cell death-1; PD-L1, programmed cell death-ligand 1; CTLA4, cytotoxic T-lymphocyte-associated protein 4; B7-1/2, peripheral membrane proteins found on activated APCs

Blockade of immune-inhibitory pathways, e.g., through inhibition of the IDO pathway, is emerging as an important modality for the treatment of cancer. However, single-agent treatments have partial anti-tumor activity in preclinical models and in cancer patients, as described above. The tumor microenvironment shows evidence of multiple immune-inhibitory mechanisms present concurrently such as PD-1/programmed cell death-ligand 1 (PD-L1) or cytotoxic T-lymphocyte-associated protein 4 (CTLA4) signaling as well as IDO signaling (Fig. 2) [82]. Remarkable clinical activity with rapid and deep tumor reduction has been reported in 65 % of 53 patients with advanced melanoma treated with a combination of nivolumab, which is different from the published data on monotherapy PD-1 inhibitor, and ipilimumab, CTLA4 inhibitor [88]. We suggest that combination immunotherapies such as IDO inhibitors with other immune checkpoint inhibitors or cancer vaccines may be required for optimal therapeutic effect. In fact, combining IDO inhibitors with anti PD-1/PD-L1 and anti-CTLA has increased the number of intratumoral CD8^+^ T cells, resulting in restored interleukin (IL)2 production [82, 89]. From the standpoint of immunology, immunotherapy is considered a ‘double-edged sword’ in that it can trigger two opposite immunologic processes. For example, among cytokines, which are major immune players, IL6 and IL10 are known to cause immune suppression or tolerance, leading to tumor progression, whereas IL2, IL12, IL23, interferon (INF)-alpha and INF-gamma are crucial in immune activation, leading to tumor suppression [90]. We may be able to better modulate the immune system by targeting both aspects: combining IDO inhibitors to block immune tolerance with immune activators such as IL2 or vaccines.

IDO inhibitors might work as single agents if IDO expression in the tumor microenvironment is the rate-limiting step and there are no other barriers to antitumor immune response, such as inadequate priming of the immune system, i.e., a lack of CD8^+^ T cells at the tumor site. Therefore, selection of biomarkers suggesting more benefit with IDO inhibitors, including expression of IDO and CD8^+^ T cells at the tumor site, should be investigated.

Another strategy to induce tumor sites to be more inflamed, or more primed for tumor immunity, is to administer targeted therapies, such as tyrosine kinase inhibitors. Frederick et al. reported that melanoma antigen expression and CD8^+^ T cells at the tumor sites were enhanced at the time of progression in patients with advanced melanoma treated with a BRAF inhibitor [91]. Interestingly, PD-L1 expression was also increased with BRAF inhibitor therapy, providing support for potential synergy of BRAF-targeted therapy and an immune checkpoint inhibitor. Further, as mentioned before, inhibition of IDO was demonstrated to contribute to the antitumor effects of imatinib in a preclinical model of gastrointestinal stromal tumor by suppressing Tregs and augmenting CD8^+^ T cells, which were partially induced by imatinib therapy [68]. A strategy of combining IDO inhibitors and various targeted therapies is strongly suggested for potential synergy.

As described above, in the phase I dose-escalating trials, IDO inhibitor monotherapy was generally very well tolerated. In addition to the excellent safety profile of IDO monotherapy, preclinical data that IDO inhibition helps overcome chemoresistance have prompted investigators to study combinations of an IDO inhibitor with other chemotherapeutic agents. As noted above, the combination of indoximod and docetaxel had a promising response rate in a preliminary report of the phase I trial [86].

## Conclusion

Preclinical and preliminary clinical data demonstrates evidence to anti-tumor activity of IDO inhibitors. However, IDO inhibitors in combination with immunotherapies, targeted agents, and chemotherapy merit thorough investigation in cancer, and more clinical trials with immune monitoring are warranted.

## Abbreviations

1-MT: 1-methyl-DL-tryptophan
ASCO: American Society of Clinical Oncology
AHR: Aryl hydrocarbon receptor
BRAF: B-Raf proto-oncogene, serine/threonine kinase
CD: Cluster of differentiation
CTLA4: Cytotoxic T-lymphocyte-associated protein 4
DC: Dendritic cells
FOXP3^+^: Forkhead box P3-positive
GCN2: General control nondepressible-2
GLK1: glucokinase
IDO: Indoleamine 2,3-dioxygenase
INF: Interferon
IL: Interleukin
mTORC1: mechanistic target of rapamycin complex 1
MLR: Mixed lymphocyte reaction
NSCLC: Non-small cell lung cancer
PD-1: Programmed cell death-1
PD-L1: Programmed cell death-ligand 1
PKC-θ: Protein kinase C theta
tRNA: Transfer ribonucleic acid
Tregs: T regulatory cells
TDO: Tryptophan-2,3-dioxygenase
TDLN: Tumor draining lymph node
